# Editorial: Metabolism Meets Function: The Multifaced Role of Metabolism in Cancer

**DOI:** 10.3389/fonc.2022.906421

**Published:** 2022-05-04

**Authors:** Isabella Giacomini, Monica Montopoli

**Affiliations:** ^1^ Department of Pharmaceutical and Pharmacological Sciences, University of Padova, Padova, Italy; ^2^ Veneto Institute of Molecular Medicine, VIMM, Padova, Italy; ^3^ Institute of Oncology Research (IOR), Bellinzona, Switzerland

**Keywords:** cancer, cancer metabolism, tumor microenviroment (TME), drug resistance, metabolic reprogramming, targeting metabolism

Cancer cells are highly proliferative cells and it has been reported that they are continuously rewiring their metabolism to support tumor growth and the enhanced energy supply.

Alterations of cancer metabolism involved several pathways, such as altered glycolysis, unbalanced lipid synthesis, or glutamine exploitation, as well as a shift toward pentose phosphate pathway or mitochondrial dysfunctions (1, 2). All these metabolic changes are known as metabolic reprogramming.

Emerging evidence reported that metabolic reprogramming of cancer cells is considered a hallmark of cancer and of drug resistance (3).

Although new discoveries in this field, there is still the need of understanding the mechanisms adopted by cancer cells that support metabolic changes, untangling the cross-link between metabolic reprogramming and tumor initiation and progression. Figuring out the molecular mechanisms that lead to alterations in cancer metabolism appears as a promising strategy for cancer therapy and to overcome drug resistance.

This Research Topic is aimed to investigate the metabolic aspects in cancer cells including (but not limited to):

- Crosstalk between metabolic reprogramming and tumor microenvironment in cancer;
- To assess whether metabolites with non-metabolic function could play a role in tumor initiation and progression;
- Characterize the phenotype of cancers establishing a correlation with metabolic reprogramming.

In addition, discussions about the promising approach of targeting metabolic alterations both alone and combined with standard therapeutic regimens will be appreciated in this Research Topic.

Authors are welcome to submit original research or review articles to provide the readers with up-to-date knowledge of the role of metabolic reprogramming in supporting and driving all the aspects of cancer biology.

## Author Contributions

IG wrote the editorial. MM conceived and edit the editorial. All authors contributed to the article and approved the submitted version.

## Conflict of Interest

The authors declare that the research was conducted in the absence of any commercial or financial relationships that could be construed as a potential conflict of interest.

## Publisher’s Note

All claims expressed in this article are solely those of the authors and do not necessarily represent those of their affiliated organizations, or those of the publisher, the editors and the reviewers. Any product that may be evaluated in this article, or claim that may be made by its manufacturer, is not guaranteed or endorsed by the publisher.
